# Src family kinases, adaptor proteins and the actin cytoskeleton in epithelial-to-mesenchymal transition

**DOI:** 10.1186/s12964-021-00750-x

**Published:** 2021-06-30

**Authors:** Maria A. Ortiz, Tatiana Mikhailova, Xiang Li, Baylee A. Porter, Alaji Bah, Leszek Kotula

**Affiliations:** 1 Department of Biochemistry and Molecular Biology, SUNY Upstate Medical University, Syracuse, USA; 2 Department of Urology, SUNY Upstate Medical University, Syracuse, USA

**Keywords:** Src family kinases, Epithelial-to-mesenchymal transition, Treatment resistance, Metastasis, Invasion, Unique domain, Actin cytoskeleton

## Abstract

Over a century of scientific inquiry since the discovery of v-SRC but still no final judgement on SRC function. However, a significant body of work has defined Src family kinases as key players in tumor progression, invasion and metastasis in human cancer. With the ever-growing evidence supporting the role of epithelial-mesenchymal transition (EMT) in invasion and metastasis, so does our understanding of the role SFKs play in mediating these processes. Here we describe some key mechanisms through which Src family kinases play critical role in epithelial homeostasis and how their function is essential for the propagation of invasive signals.

**Video abstract**

**Supplementary Information:**

The online version contains supplementary material available at 10.1186/s12964-021-00750-x.

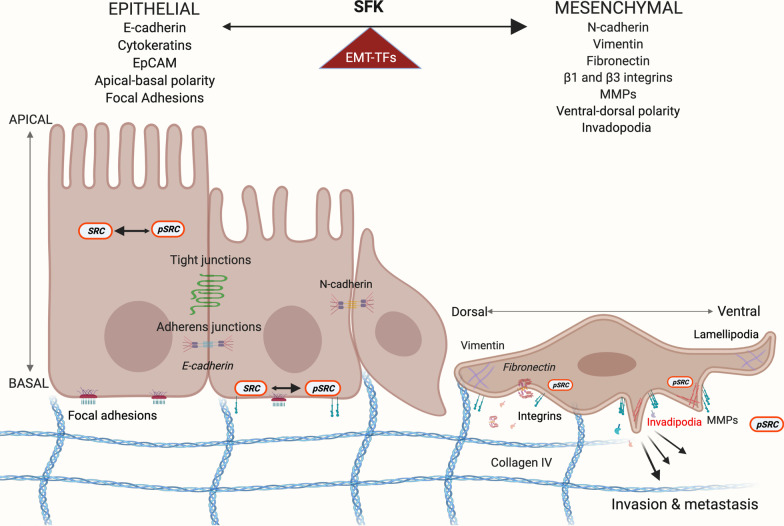

## Background

SRC is the transforming product of the first identified oncogenic virus and the prototype for SRC family kinases (SFKs). Pioneering work conducted by Peyton Rous in 1911 on viral SRC (v-SRC), sparked years of research seeking to understand SRC-driven oncogenesis [1, 2]. Initial observations showed that SRC activity was positivetily correlated with cancer progression into a metastatic state. This prompted the idea that SRC function may be essential for the development of metastasis and invasion [3]. Since then, SFKs have been identified as key players in tumor progression, invasion and metastasis in a variety of different cancers.

Currently, the mainstay hypothesis which explains the cellular processes involved in the regulation of invasiveness and metastasis is the epithelial-to-mesenchymal transition (EMT). The EMT refers to a cellular program that allows epithelial cells to obtain mesenchymal features, which has been shown to generate more invasive and treatment-refractory cancers. While EMT is important in a variety of processes such as embryonic development (Type I EMT) and wound healing (Type II EMT); this review will focus on EMT in carcinogenesis, metastasis and invasion (type III EMT) [4]. Moreover, it is important to note that while EMT in embryogenesis results in a fully differentiated mesenchymal state, in the context of carcinogenesis, partial EMT is most commonly observed giving rise to cancer cells that exhibit both epithelial and mesenchymal features [5, 6]. With the evergrowing body of literature supporting the role of the epithelial-mesenchymal transition (EMT) in invasion and metastasis, so does our understanding of the role SFKs play in mediating these processes [7]. Here, we describe the mechanisms through which SFKs regulate epithelial homeostasis and how SFK dysregulation is key in promoting the EMT in various cancer. Moreover, we will explore the role of SFKs in mediating treatment resistance and the implications of this resistance for the future of SFK inhibitors in the treatment of metastatic diseases.

### Src family kinases

**Fig. 1:**
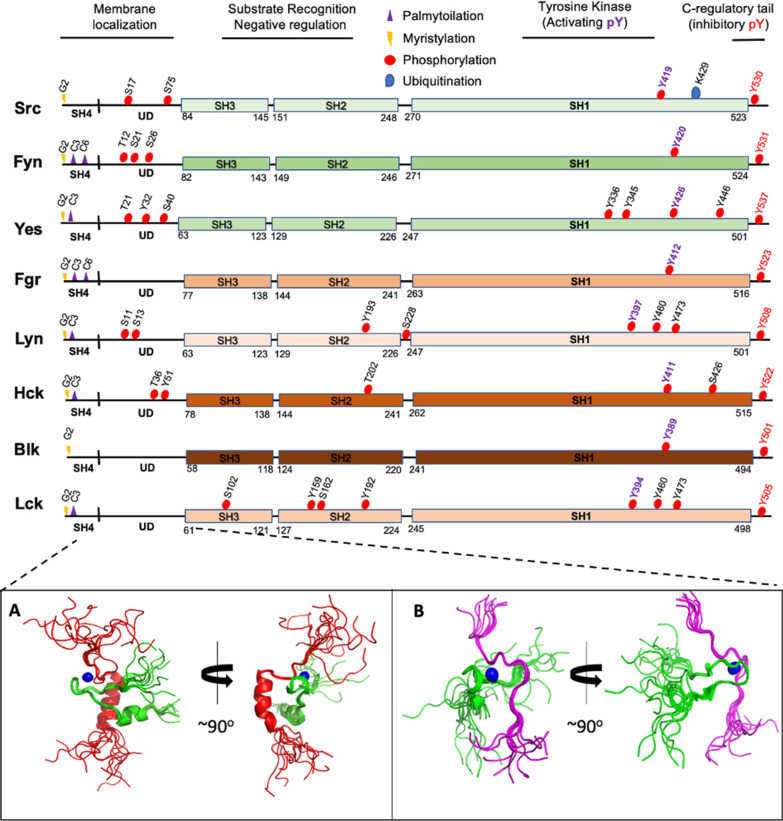
Primary structure of SRC family kinases. Domain and signal conservation within Src family kinases. Src family kinases are activated at the membrane, which involves lipid/myristate modification within the SH4 region and membrane binding. There is very little known about the UD, which might be also involved in membrane localization, activation and ligand substrate binding (see lower panel for LCK). SH3 and SH2 domain bind substrates and regulate the catalytic activity of the tyrosine kinase (domain). Posttranslational modifications in Src family kinases essential for membrane localization (myristylation and palmytoilation); activation (within kinase domain) and inhibition (C-terminal tail) (phosphorylation) are highlighted in the diagram. Domain/regulatory regions are depicted as lines and boxes: Src homology 1, SH1, tyrosine kinase/catalytic domain; SRC homology 2 or SH2; SRC homology 3, SH3; SRC homology 4, SH4, and unstructured Unique Domain, UD). Bottom panels: NMR Structural Ensemble of the C-terminal tail of (**A**) CD4 (red) or (**B**) CD8α (magenta) in complex with the intrinsically disordered Unique Domain of LCK (residues 7–35). Both complexes are very dynamic and are mediated by Zn (blue spheres). The pdb codes are 1Q68 and 1Q69 for CD4 and CD8α, respectively

Src family kinases (SFKs) are non-receptor tyrosine kinases involved in the regulation of important cellular functions such as cell proliferation, differentiation, apoptosis, migration, and metabolism [8, 9]. The vertebrate Src kinase family is composed of nine members, namely, SRC, LCK, LYN, BLK, HCK, FYN, FGR, YES, and YRK. Please note that YRK is only expressed in chickens, so we will omit it in subsequent sections in order to focus on the role of SFKs in human cancers. It is curious that the YRK protein kinase in chickens is 95% identical to the FGR protein kinase in black swans. Based on the prolife of YRK expression, the *Yrk* gene seems to be very similar to the FGR gene in vertebrates (Fig. 1, top panel). Moreover, initial characterization of SFKs included tyrosine kinases such as SRM and FRG, however, amino acid sequence and intron/exon structural analysis placed these kinases in the Brk kinase family [10, 11].

### SFK structure, localization and function

Src family kinases are modular proteins that share a common domain architecture consisting of both intrinsically disordered regions (IDRs) and folded domains. At the N-terminus is an intrinsically disordered membrane-anchoring, myristoylated Src homology domain 4 (SH4), followed by regulatory folded domains SH3 and SH2, as well as an enzymatically active tyrosine kinase domain (SH1) connected to an intrinsically disordered C-terminal regulatory region [8, 9, 12, 13]. The SH3 and SH4 domains are linked by another IDR termed Unique domain (UD) [14, 15]. The structure and functions of the folded SH3, SH2, and SH1 kinase domains of SFKs have been extensively studied in exquisite detail [9]. However, in the past two decades, emerging structural and biochemical studies have also started to elucidate the crucial role of the UDs, and the synergy of the UD and the folded domains, in the regulation of SFK subcellular localization and activity [8, 16–18]. Unlike the folded domains that are highly conserved across the whole Src kinase family, the UDs of the various SFK have quite variable lengths and amino acid sequence composition [8, 14]. However, the UDs of the individual kinases are surprisingly highly conserved among species and to demonstrate the physiological importance of the UD in SFKs, previous studies show that swapping the UD of SRC and YES interchanges their functional specificity [19–21]. The UD of ﻿SRC, which has been the ﻿subject of the most intense structural, biophysical and biochemical investigations, is shown to ﻿adopt a compact, yet highly dynamic, intramolecular fuzzy structure that uses the SH3 domain as a scaffold [22]. However, further studies by Miguel Pons and colleagues suggests that, despite their lack of conservation, the UDs of the SFKs shares a common mechanism for connecting the disordered and structured domains within the SFKs. Indeed, the existence of multiple post-translational modifications, such as phosphorylation, that modulate the biologically relevant inter-and intra-molecular interactions of UDs of SFKs reinforces the critical functions played by the intrinsically disordered UD in regulating the localization and activities of SFKs [8, 22].

**Fig. 2:**
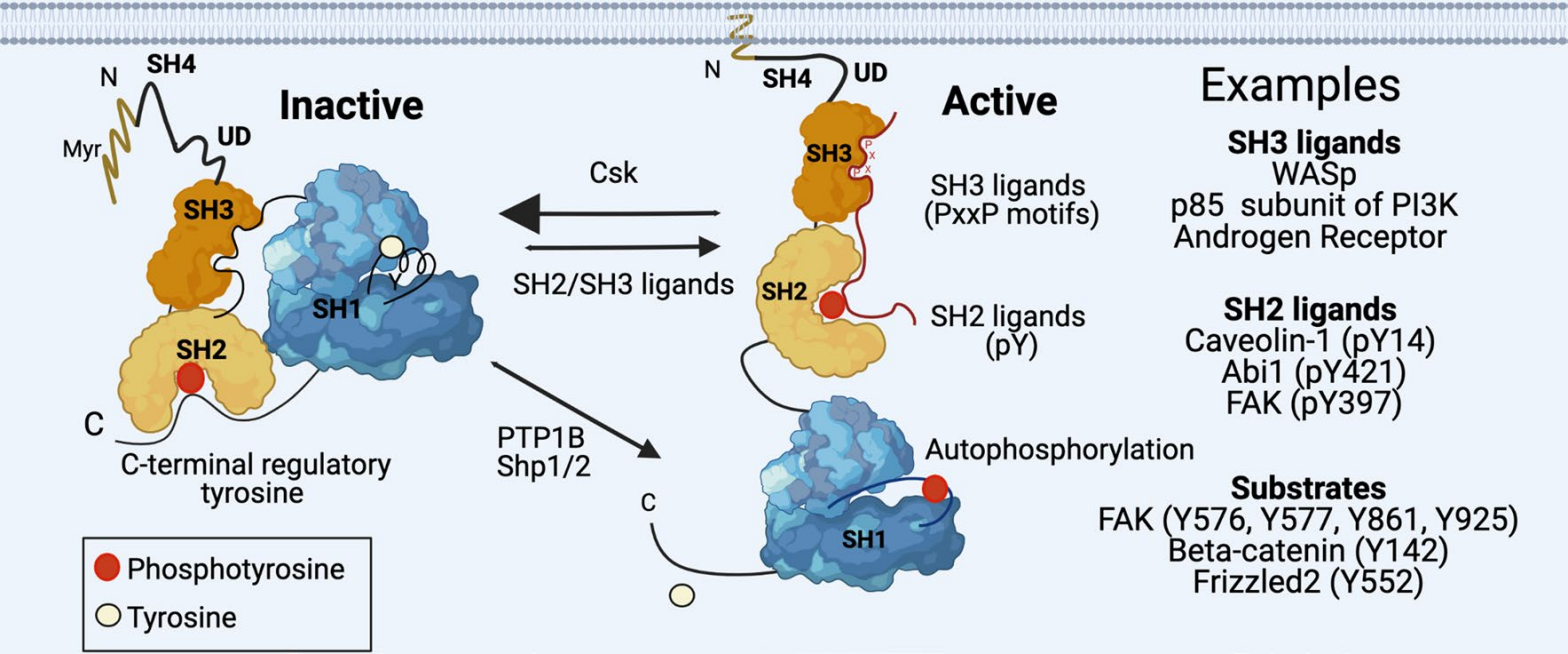
Conformational changes associated with Src family kinase activation and inhibition. Left, inactive conformation of Src family kinases is associated with lack of membrane binding, lack of phosphorylation of the activation loop tyrosine, phosphorylation of the C-terminal regulatory region tyrosine; and characterized by a “closed conformation”. The closed conformation is maintained through inhibitory SH3-SH2 domain interactions with the catalytic domain (SH1), and the C-terminal regulatory phosphotyrosine interaction with the SH2 domain. These interactions prevent ligand substrate binding. Right, Active “open” conformation allows for ligand binding and autophosphorylation of the activation loop tyrosine. The balance of active/inactive conformation is regulated by Csk that promotes phosphorylation of C-terminal tyrosine; and its dephosphorylation by PTP1B/Shp1/2. Ligand binding facilitates activation but may also regulate kinase activity through restricting access (competitive inhibition) to the active catalytic domain. Examples of SRC kinase ligands/substrates are listed on the far right. Created with BioRender.com

Regulation of membrane localization is a critical mechanism governing the biological functions of SFKs [23]. Thus, cells have evolved multiple interdependent mechanisms, which are mediated via intra- and intermolecular interactions, for regulating of SFK subcellular localization to the plasma and intracellular membranes; to the cytoskeleton, signaling foci, subcellular compartments and organelles as well as to the extracellular matrix [16, 23, 24]. First among these regulatory mechanisms is a critical N-terminal sequence (Met-Gly-X-X-X-Ser/Thr) in the SH4 domain that allows for irreversible co-translational attachment of a myristate group—14 carbon saturated lipid [25]. Before translated protein products are released from the ribosome, a myristic acid-derived myristoyl group is attached to the N-terminal glycine by N-myristoyltransferase enzymes. This modification allows water-soluble proteins to associate with hydrophobic membranes. In addition to myristoylation, membrane targeting of SFKs is further facilitated by the presence of positively charged residues that allow for electrostatic interaction with negatively charged head groups of the membrane phospholipids (Fig. 2). Both of these structural components are essential for membrane anchoring as their alterations are associated with altered SFK-mediated cell signaling [26].

Another important membrane-anchoring mechanism that utilizes post-translational modification which occurs at the SH4 domain of many SFKs is palmitoylation—a reversible attachment of deprotonated fatty acid group to a cysteine residue. It turns out, palmitoylation is dependent on myristoylation, and while all SFKs are myristoylated, SRC and BLK lack the N-terminal cysteine necessary to complete the myr-Gly-Cys motif required for palmitoylation [25, 27, 28]. Interestingly, the interaction of SFKs with lipid membranes is not restricted to the post-translational modifications (PTMs)-facilited anchors and amino acid sequence composition of the SH4 domain alone. In 2013, Pérez et al. discovered that a partially structured region within the UD of SRC, aptly named unique lipid binding region (ULBR), that can interact with lipids in a T37 and S75 phosphorylation-dependent manner [16]. Moreover, there is evidence suggesting the existence of a coupling mechanism between the lipid binding properties of the ULBR and SH4 domain binding. Furthermore, members of the SFKs can use their modular domain architecture to localize to plasma membranes and intracellular membranes via protein:protein interactions with membrane-anchored receptors. For instance, LCK utilizes its UD to mediate its association with the intrinsically disordered tails of the cell membrane-anchored T cell co-receptors CD4 or CD8α by undergoing a disorder-to-order transition to form a compact heterodimeric zinc-mediated complex (Fig. 1, bottom panel) [29]. In addition, Salter and colleagues have also demonstrated that NADH dehydrogenase subunit 2 (ND2) anchors SRC kinase to N-methyl D-aspartate receptors (NMDARs) at post-synaptic densities (PSDs) in the hippocampus via an interaction involving SRC UD residues 40–49 and ND2 residues 239–321 [30, 31].

Following the multifunctional, intrinsically disordered UD are the folded reguratory SH3 and SH2 domains, which can also be used to anchor SFKs to membrane-bound and non-membrane-bound signaling complexes through interactions involving binding partners with proline-riched and phosphotyrosine-containing motifs, respectively [32, 33]. Interestingly, these regulatory SH3 and SH2 domains are also found in many other signaling proteins, where they are utilized to help assemble membraneless, liquid–liquid phase separated organelles via transient multivalent interactions [34].

Next, we will briefly describe the structure and binding mechanisms used by these regulatory domains to control the kinase activity of SFKs. SH3 domains are small, approximately sixty amino acid-residue folded β-barrels, consisting of five antiparallel strands that mediate protein:protein interactions by binding to proline-rich sequences that can adopt a left-handed helical conformation [35]. SH3 domains contain two prominent loops, known as the RT and n-SRC loops, that lie at either end of a surface generated by aromatic and hydrophobic residues that make up the recognition site for PxxP motifs. For instance, as part of the regulatory intramolecular interactions in SFKs, ﻿the linker between the SH2 domain and SH1 kinase domain contains a proline residue that interacts with the SH3 domain, thereby stabilizing the ‘closed’ inactive conformation (Fig. 2) [9, 36].

In contrast, the SH2 domains are modular phosphotyrosine-binding domains that consists of an approximately 100 amino acid residues, which fold into structure with two α-helices (a1 and a2) that packed against each side of a central three-stranded β-sheet [37]. The SH2 domain structure has a conserved argine-containing recognition pocket for binding phosphotyrosine residues and another pocket for binding hydrophobic residues C-terminal to the phosphotyrosine motif. Interestingly, SH2 domains can bind to a variety of phosphotyrosine-containing sequences that do not contain the consensus pYEEI motif, because residues well beyond the vicinity of the phosphotyrosine modification also contribute significantly to the extended binding interface. One predominant mechanism for controlling the kinase activity of SFKs is the formation of an intramolecular interaction between the SH2 domain and a pTyr residue located at the intrinsically disordered C-terminal regulatory region, resulting in a ‘closed’ inactive conformation [9, 38]. Hence, these conformational states are regulated by the balanced activity of the kinase Csk, promoting the inactive conformation, and phosphatases such as ﻿PTP1B and Shp1/2, which promote displacement of this interaction due to dephosphorylation of this phosphotyrosine. In addition, competitive binding from signaling proteins containing phosphotyrosine motifs, lead to an ‘open’ active conformation of SFKs (Fig. 2). ﻿In another major activation mechanism, SRC and other SFKs can undergo intermolecular autophosphorylation of a Tyr residue located at the activation loop of the SH1 catalytic domain to enhance kinase activity. The SH1 domain, which is the catalytic center of the kinase, contains two ‘lobes’: a small lobe found in all protein kinases is generated by a five-stranded antiparallel β-sheet and an important regulatory αC-helix, while the ﻿large lobe of the kinase domain consists of mainly conserved α-helical segments. ﻿The active site of the kinase domain lies within a cleft nestled between the small N-lobe and the large C-lobe. As described above, there are multiple intra- and intermolecular regulatory mechanisms that control substrate specificity and activity of SFK kinase domain. These mechanisms include destabilization of the intramolecular interactions involving the SH3 and SH2 domains by phosphatases, kinases, scaffolding proteins, and substrates with PxxP- and/or phosphotyrosine-containing motifs that shift the conformational equilibrium to an ‘open’ active state (Fig. 2) [9, 38].

**Fig. 3:**
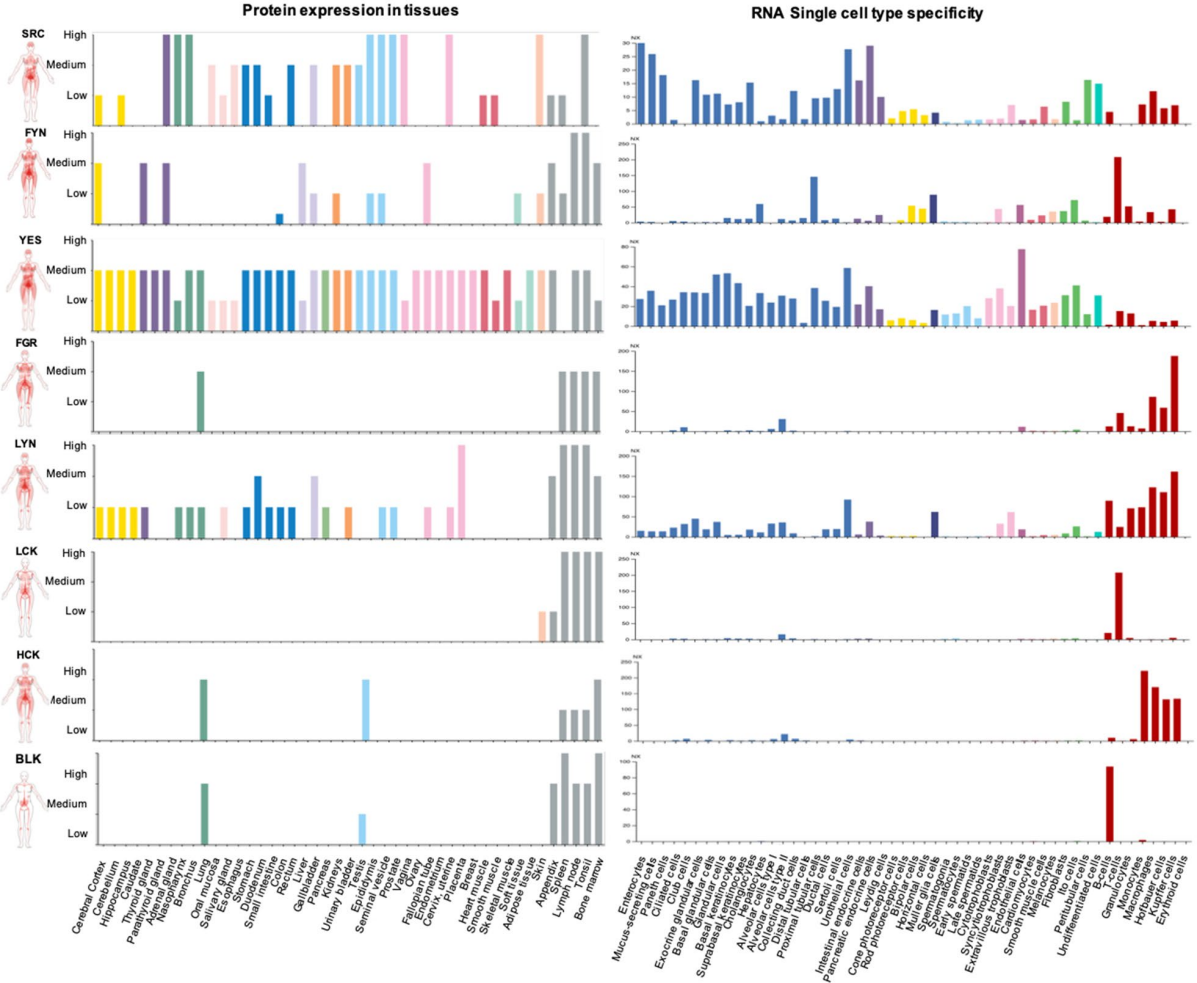
Differential expression of Src family kinases at the tissue and cellular levels. Left panel, Protein Atlas aggregate data on expression pattern of Src family kinases in tissues. Based on these information four kinases, SRC, FYN, YES and LYN, can be considered ubiquitously expressed. While FGR, LCK, HCK, and LYN are more restricted to tissue associated with immune response and blood cell production (bone marrow and spleen), and lungs. Right panel, RNA-based single cell type expression data of SRC family kinases. These data also support general expression patterns of SFKs. Human Protein Atlas available from http://www.proteinatlas.org (Tissue atlas “protein expression overview” and cell type atlas “single cell types”; Gene entries: *SRC*, *FYN*, *YES1*, *FGR*, *LYN*, *LCK*, *HCK*, *BLK*)

In addition to controlling their membrane localization and activities at the protein level, the function of SFKs is also regulated by controlling their transcriptional and translational expression in various tissues, and cell-types (Fig. 3) as well as their differential sorting to various subcellular compartments and the extracellular matrix. For instance, SRC, FYN, LYN and YES are widely expressed, while other members have a more restricted expression pattern, especially to cells of hematopoietic origin [39, 40]. Furthermore, expression levels of SFKs vary among cell types, for example, while SRC is ubiquitious, its expression is highest in platelets, neurons, and osteoclasts (Brown & Cooper 1996).

Moreover, coexpression of different SFKs may vary among cellular subtypes and subcellular compartments (Fig. 3). Work by Kuga and colleagues showed that while SRC, YES and LYN are all co-expressed in HeLa cells, they were activated at different levels by the cyclin-dependent kinase 2 (Cdc2) during the M phase of mitosis [40]. As discussed above, subcellular localization plays a critical role in the function of SFKs. Indeed, SFKs have been found in a variety of different subcellular compartments such as caveolae, focal adhesions, endosomes, lysosomes and nucleus [41–45]. In addition to its established intracellular functions, there is emerging evidence showing that the prototypic SFK, SRC, also has extracellular functions such as regulation of MMP2 activity through phosphorylation of its inhibitor, the tissue inhibitor of metalloproteinases 2 (TIMP-2) [46]. A recent study by Tanaka et al., show that SRC extracellular secretion was regulated by ubiquitination at Lys429, a highly conversed residue among all SFK members, suggesting this mechanism may also be applicable to other SFK members [47]. Taken together, these multiple regulatory mechanisms demonstrate that there is an intricate cellular network of lipid-modifying enzymes, kinases, phosphatases, binding partners and substrates from diverse signaling pathways that coordinate to tighly control the localization and to fine-tune the level of SFK catalytic activity. Thus, like in other modular proteins [48], the functions of the IDRs and folded domains within the SFKs synergize to precisely control the biological functions of SFKs. However, when that synergy is disrupted due to mutagenesis of the cognate proteins involved or hijacked by disease-causing agents, devasting maladies like cancer can result [49].

## Src family kinases in the epithelial mesenchymal transition (EMT)

Src family kinases have been shown to interconnect a variety of different cellular pathways which promote invasion and metastasis. Unsurprisingly, these pathways are also critical in the propagation and initiation of the epithelial-to-mesenchymal transition [7]. The epithelial-to-mesenchymal transition (EMT) is a cellular program which enables epithelial cells to acquire mesenchymal features and has been shown to be critical in embryonic development, wound healing, and malignant progression [50]. Moreover, the EMT has been linked to acquisition of treatment resistance in a variety of malignancies including solid tumors and hematologic malignancies, all of which underscore its clinical significance [51, 52]. Interestingly, the stromal tissue which surrounds a carcinoma is not much different than that of stroma in non-tumorigenic tissue undergoing healing and/or inflammation. This has raised the question of whether these are distinct processes or the same process occuring under physiological versus pathological environments [6]. However, it is important to note that EMT in carcinogenesis refers to a transient and malleable cellular program which can be altered and reversed through activation of the mesenchymal-to-epithelial transition (MET), a process believed to be critical for colonization of metastatic sites [53]. Unlike tumorigenesis which occurs as a result of a cell-autologous insult (i.e. oncogenic mutations, tumor suppressor loss), EMT is an inducible process which results from a combination of epigenetic alterations and tumor-stromal interactions that lead to transcriptional reprogramming through activation of EMT transcription factors (EMT-TFs) [6]. Hence, in the following sections, we will evaluate the role of SFKs in mediating the morphological, signaling, and transcriptional alterations associated with the EMT.

### SFKs and the actin cytoskeleton

**Fig. 4:**
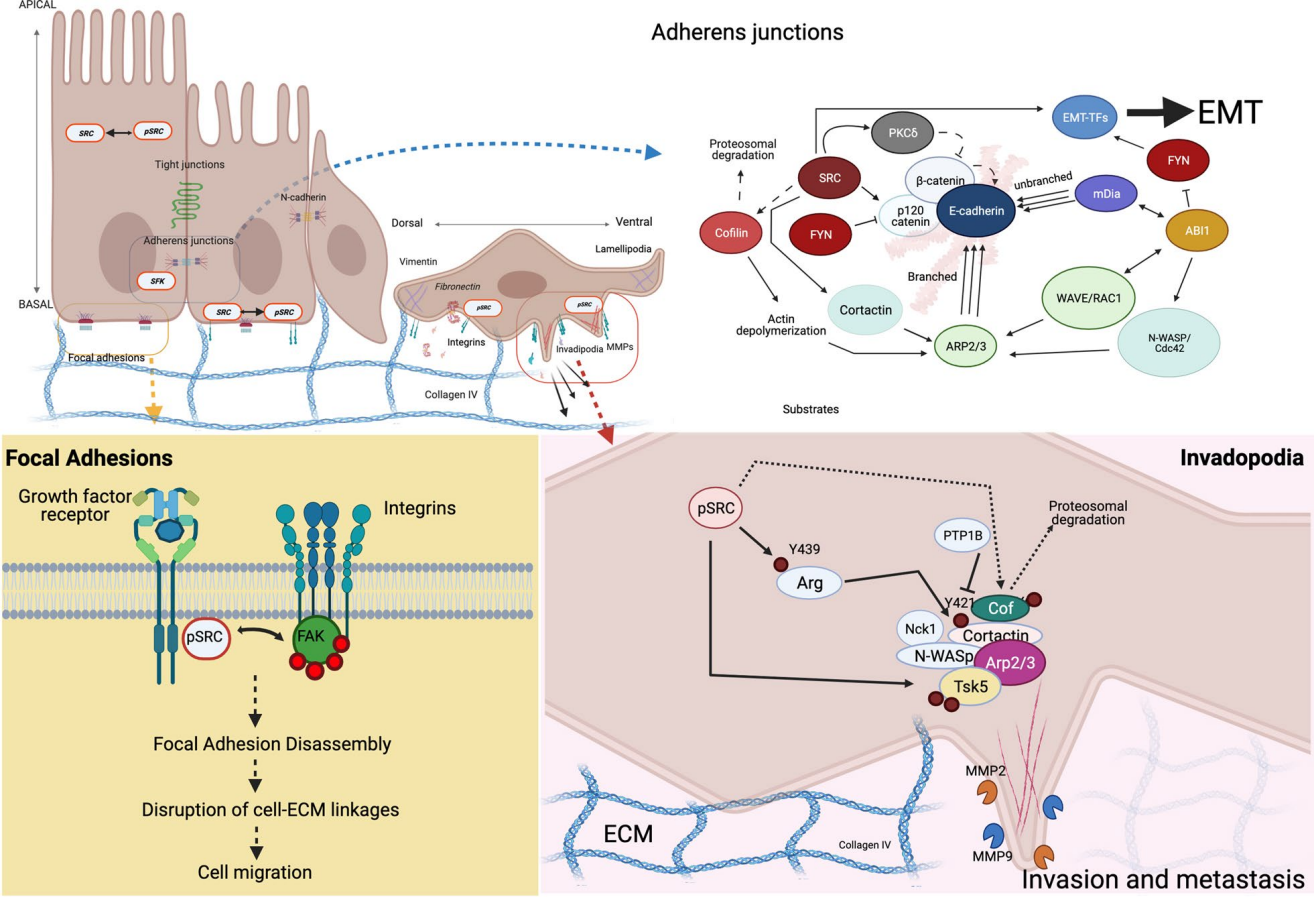
Cell structures critical for epithelial homeostasis and regulated by the interface of SFKs and actin cytoskeleton regulatory complexes. Loss of integrity the structures and /or deregulation of these complexes promote EMT. Top left, Major cell adhesion and invasive phenotypes mediated by SFKs critical for the epithelial to mesenchymal transition. Top right, Cell–cell adhesion, with adherens junctions regulated by E-cadherin, catenins, p120. WAVE complex regulates actin cytoskeleton input into adherence junctions with ABI1 being a key adaptor protein for SRC at the membrane (see text for more details). Lower left, Active SRC regulates FAK to maintain and regulate integrin function. Integrins are intimately involved in cell migration, invasive potential and tissue specificity during metastasis. Lower right, a key complex that regulates invadopodia through SRC-Arg and SRC-Tsk5 axes: N-WASp, Arp2/3, cortactin and control actin polymerization input in invadopodia. MMP2 and MMP9 degrade collagen and other ECM integral proteins. Created with BioRender.com

SFKs are key regulators of morphology and epithelial integrity through regulation of actin cytoskeletal dynamics and cellular adhesions. The EMT is characterized by morphological changes which occur as a result of disappearance of cell–cell junctions and actin-cytoskeleton rearrangement [50]. Changes in other cytoskeletal proteins like intermediate filaments also occur, with the most important one being a decrease in cytokeratin and upregulation of vimentin [54]. Early studies showed SRC-induced mitosis and morphological changes were driven by separate pathways; however, it was also noted that both required membrane localization [55]. Specifically, work by Frame et al., showed that SRC activity was spatiotemporally dependent: at earlier times upon activation oncogenic SRC (v-SRC) localized to the periphery predominantly to discrete adhesions and relocated to the membrane and residual adhesion at later times [55]. Changes in E-cadherin expression during the EMT are largely attributed to hypermethylation and transcriptional repression of the E-cadherin gene (*CDH1*) mediated by EMT-TFs. Classical EMT transcription factors such as Snail and ZEB1 bind directly to the *CDH1* promoter and repress its transcription. Moreover, EMT-TFs have been shown ro recruit other transcriptional regulatory complexes to the *CDH1* promoter. For example, both SNAIL and ZEB1 repress E-cadherin expression through direct binding to the E-boxes in the *CDH1* promoter, with SNAIL recruiting the polycomb repressive complex [56–58], while ZEB1 recruits chromatin modifier complexes [59, 60]. E-cadherin repression leads to cellular junction disassembly, loss of typical epithelial cobblestone morphology and acquisition of mesenchymal spindle-shaped morphology [6]. However, while the formation of new E-cadherin is repressed transcriptionally by EMT-TFs, E-cadherin present in cellular junctions needs to be destabilized in order for adherens junctions to disassemble. Early work by Behrens et al., linked SRC activation to increased E-cadherin/β-catenin phosphorylation, loss of cell–cell contacts and acquisition of fibroblast-like morphology [61]. Thus, in the next sections we will discuss the role of SFKs in mediating key morphological and adhesion changes associated with the EMT, with a particular focus on those dependent on actin-cytoskeleton dynamics (Fig. 4).

#### Changes in cell adhesion and polarity

A key feature of the EMT is loss of cellular junctions such as adherens junction (AJ) and focal adhesions (FA). Adherens junctions link adjacent epithelial cells together, as such, they play a critical role in epithelial cell integrity, tissue formation and tumor suppression [62]. AJs consist of cadherin-based cellular junctions attached to the actin-cytoskeleton by catenin proteins. Briefly, E-cadherin is an adhesion molecule that forms homodimers with molecules on adjacent cells. On the other hand, catenins serve to stabilize cadherin-based junctions by linking them to the actin cytoskeleton. Specifically, p120-catenin binds to the cytoplasmic tail of E-cadherin and stabilizes it by inhibiting its endocytosis; β-catenin also binds to E-cadherin and recruits α-catenin; then, α-catenin binds to actin and recruits other important actin-binding proteins which together regulate junctional stability [63–66]. As such, regulatory complexes involved in actin-cytoskeleton dynamics play a critical role in the stabilization and formation of adherens junctions, many of which have been implicated with invasion and metastasis in a variety of different cancers [66].

The ARP2/3 complex is one of three known actin-nucleators and it is unique in its ability to organize branched-actin filaments [67]. The most well characterized nucleation-promoting factors (NPF) known to activate ARP2/3 activity are Wiskott–Aldrich syndrome protein (WASP and neural (N)-WASP) and WAVE (WASP-family verprolin-homologous protein), which function downstream of small RHO family GTPases [67]. WAVE is a heteropentameric complex comprised of WAVE 1/2/3, ABI1/2/3, CYFIP1/2, NAP 1/2, and BRK1(HSPC300), which regulates ARP2/3-dependent actin dynamics through activation of the Rho family small GTPase, RAC1 [68]. Work by Takenawa and colleagues showed the essential role of the WAVE regulatory complex (WRC) in the formation and stabilization of AJs [68]. Interestingly, authors showed that depletion of the adaptor protein ABI1 or BRK1 had a more significant effect on cell–cell adhesion formation than did WAVE2 depletion, which was attributed to the redundancy between WAVE1 and WAVE2 proteins, as double knock-down of WAVE1/2 generated a more pronounced defect on cell–cell junction formation than either alone [68]. Later, it was shown that ABI1 was also involved in regulating unbranched actin-polymerization by a formin-dependent pathway through interactions with the Diaphanouse formin, mDia1 [69]. Moreover, ABI1 also binds to and activates N-WASP/Cdc42-dependent Arp2/3 activation [70]. Of note, in addition to its Abl-kinase regulatory role through pY213, previous work from our lab has shown ABI1-pY421 is a high affinity substrate for the SH2 domain of SFKs [71, 72]. More recently, we showed that ABI1 is a key EMT regulator through modulation of the WNT5a-FYN-STAT3 signaling axis in prostate cancer [73]. Combined, these findings show that ABI1 is a key connector of actin-cytoskeletal dynamics through the regulation of kinases and actin nucleation promoting factors (Fig. 4, Top right panel).

Work conducted by Chen and colleagues showed that SRC-mediated PKCδ-activation in response to growth factor stimulation led to increased phosphorylation of E-cadherin at Thr790, which decreased its binding affinity for β-catenin and promoted the dissociation of adherens junctions [74]. Castaño et al., showed the importance of SFK-mediated phosphorylation of p120-catenin-induced Rho A activation. While FYN-mediated phosphorylation of Y112 inhibited p120-catenin interaction with Rho A, SRC-mediated phosphorylation of Y217 and Y228 promoted this interaction [75]. Moreover, Yoo et al., showed that v-SRC-mediated phosphorylation of cofilin at Y68 induced its ubiquitination and proteosomal degradation [76]. Cofilin is a key regulator of actin-dynamics and membrane protrusions by promoting actin-depolymerization, and generates preferred substrates for Arp2/3-mediated actin polymerization [77]. Hence, SFKs play a critical role in modulating the activity of both actin-polymerization and depolymerization at adherens junctions. This suggests that cellular junction stability is tighly regulated by the interplay between different SFKs.

In addition to disruption of cellular junctions, EMT activation induces changes in cell-polarity through disruption of tight junction protein complexes involved in cell polarity maintenance [78, 79]. Typically, epithelial cells have apical-basal polarity, which gets converted to front-rear polarity in the mesenchymal state. Partitioning-defective (PAR) complexes (comprising PAR6, PAR3 and atypical protein kinase C (aPKC) and Crumbs complexes (comprising Crumbs (CRB), protein associated with Lin-7 1 (PALS1) and PALS1-associated tight junction protein (PATJ) localize apically in association with tight junctions and define the apical compartment. Scribble complexes (comprising Scribble (SCRIB), Discs large (DLG) and lethal giant larvae (LGL)) define the basolateral compartment. This is also accomplished through reorganization of the actin cytoskeleton. Combined, these morphological and polarity changes prepare the cell for acquisition of motility. SRC promotes cellular junction disassembly through activation of the focal adhesion kinase (FAK). Once activated, FAK promotes focal adhesion disassembly, disrupts cell-ECM linkages and initiates cell migration through regulation of integrin signaling [80].

#### Invadopodia

SRC has been shown to localize primarily to the membrane of invading tumors. Cells acquire invasive properties through formation of actin-based protrusions known as invadopodia along with secretion of matrix metalloproteinases (MMPs), which can degrade extracellular matrix (ECM) proteins [81]. Invadopodia are specialized membrane protrusions containing a primarily branched F-actin core and actin regulatory proteins, induced by growth factor or ECM signals [82, 83]. Many of these growth factor invadopodia-inducing pathways converge on key signal transducers like Rho family GTPases, phosphoinositide 3-kinase (PI3K) and SRC kinase [84, 85]. Work conducted by Artym et al., showed that there are two aspects to invadopodium, a structural and a functional component [83, 86].

The structural component recruits actin-regulatory complexes such as N-WASP, cortactin, Arp2/3, and dynamin [87]. Many essential components for invadopodia formation have been shown to interact with and/or be phosphorylated by the prototypic SFK, c-Src. Cortactin is another important Arp2/3 nucleation promoting factor and is also a SRC-substrate [88]. Cortactin’s activity promotes and stabilizes branched-actin filament formation by directly binding to the Arp2/3 actin-regulatory complex [89]. SRC-mediated phosphorylation of cortactin at Y421 enhanced actin assembly, and has been shown to be critical in mediating activation of the Nck-N-WASp complex, one of the earliest steps in invadopodia formation [83, 90, 91]. Furthermore, SRC-mediated phosphorylation of Tsk5, an adaptor protein essential for invadopodia formation, has been shown to regulate invadopodia-associated invasion in prostate cancer [92, 93].

As previously mentioned, the functional component of invadopodia utilizes matrix metalloproteases (MMPs), which are proteolytic enzymes that degrade the ECM [86]. Invadopodia have been shown to rely on membrane type 1 MMP (MT-MMP), MMP-type 2 (MMP-2) and MMP-9 for ECM degradation [94, 95]. MMP-2 and MMP-9 are particularly linked to invasion and metastasis as they are able to degrade collagen type-IV, a major component of the basement membrane [96]. Previous work conducted by Cortes-Reynosa and colleagues showed that MMP-9 secretion was mediated in a SRC and FAK-dependent manner [97]. Moreover, Eckert and colleagues showed that SRC, activated through TWIST1 transcription factor and platelet-derived Growth Factor Receptor (PDGFRα), mediates formation of invadopodia to degrade ECM and promote cancer cell invasion. Treatment with selective SRC inhibitors reduced ECM degradation by fivefold [82]. Moreover, Abelson-interactor 1 (ABI1), a WAVE regulatory member and N-WASP regulator, has also been implicated in the regulation of invadopodia formation through regulation of the SRC-Id1-MMP9 axis [98]. All of these combined support SRC’s critical role in invadopodia formation.

### SFK’s role as signal transduction mediators in EMT-inducing pathways

**Fig. 5:**
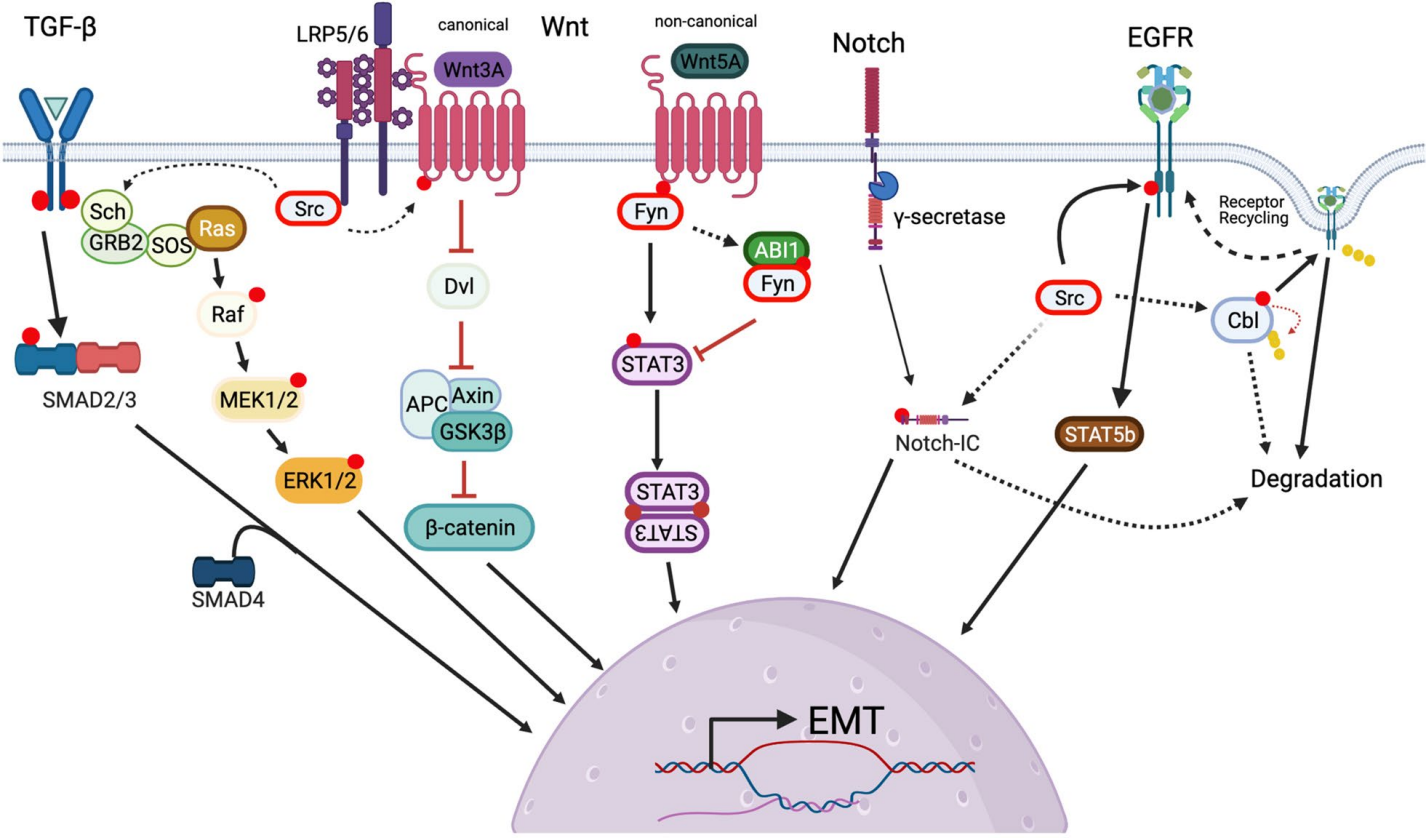
Cell signals that regulate SFKs input into major cellular pathways. Roles of SFKs in modulating some of the key EMT-inducing pathways. Top, Signals initiated by a variety of receptors, TGF-beta, WNT pathway receptors (LRP5/6), Notch and growth factor receptors such as EGFR. All signals involve cytoplasmic regulators and modulators which end up activating transcription factors/coactivators to regulate gene expression. See text for details. Created with BioRender.com

The classical EMT-TFs include the zinc-finger E-box binding homeobox factors ZEB1 and ZEB2, Snail (SNAI1), SLUG (SNAI2), and the basic helix-loop-helix factors TWIST1 and TWIST2 [99]. However, other transcription factors have been identified to be critical in the progression of EMT in different tumors [100, 101]. A feature which strengthens the robustness of the EMT once initiated is the positive feedback among EMT-TFs. For example, SNAIL enhances WNT signaling by improving the transcriptional activity of β-catenin via direct interactions in the nucleus and by increasing responsiveness to WNT signaling. As a transcriptional cofactor, β-catenin binds to the promoter region of other EMT-TFs and induces their expression (102, 103). This reinforces the idea that EMT in carcinogenesis results from an interplay between driver-mutations and hijacking of developmental pathways which allows cancer cells to overcome extrinsic insults (e.g. host-immunity, anti-cancer therapeutics), invade and metastasize. SFKs have no intrinsic transcriptional activity, however, they have been shown to directly phosphorylate and activate the transcriptional activity of EMT-inducing transcription factors such as the androgen receptor (AR) and the signal transducer and activator of transcription 3 (STAT3) [73, 104–106]. This emphasizes that while EMT-TFs are critical in maintaining global cellular changes through regulation of gene expression, alterations in signaling pathways are responsible for inducing and executing these changes. SFKs have been shown to be critical in a variety of signaling transduction pathways, known to regulate the EMT, in which they serve as both inducers and connectors. Moreover, dysregulated signaling pathways may be the cause for the partial-EMT observed in cancer which results in tumor cells displaying characteristics that fall under a spectrum of epithelial and mesenchymal features rather than a fully differentiated state. Thus in the following sections we will evaluate the roles of SFKs in modulating activity of some key EMT-inducing pathways. Specifically, TGF-β/SMAD, Wnt, NOTCH and EGFR signaling (Fig. 5).

#### TGF-β pathway

TGF-β signaling has been heavily linked to the activation of EMT and cancer stem cell (CSC) generation. TGF-β lingands bind to complexes of TGF-β receptor Type 1 (TGFβR1) and TGFβR2, leading to the phosphorylation of SMAD2/3, which proceed to form heteromeric complexes with SMAD4. Similarly, engagement of bone morphogenic proteins (BMPs) lead to activation of SMAD1/5, which can also complex with SMAD4. Once these SMAD trimeric complexes migrate to the nucleus, they activate transcription of mesenchymal genes. TGF-β signaling induces the formation of myofibroblasts that secrete significantly higher levels of TGF-β which helps induce and/or maintain EMT in adjacent carcinoma cells. In addition to signaling through cytokine and growth factors, cancer associated fibroblasts can alter methylation patterns in EMT-regulating genes and induce mesenchymal features and CSC-like properties. Shipitsin et al., showed that there is an increase in TFGβ -1 and TFβII in CSC when compared to more differentiated controls. Additionally, inhibition of TGF-β signaling led to acquisition of a more epithelial phenotype [107].

Multiple studies support the role of SRC in inducing the transition of TGFβ signaling from tumor suppressive to oncogenic. Work by Galliher et al., identified the role of the αvβ3/SRC/TβRII signaling axis in promoting the oncogenic signaling of TFG-β. Specifically, β3 interacts with TGFβR2 which allows SRC to phosphorylate TβR-II on Y284, generating a docking site for the SH2 domains of Grb2 and Shc. Abrogation of this phosphorylation site in TβR-II did not affect SMAD2/3 activation but completely prevented p38 MAPK activation [108]. More recently, Zhang et al. showed that TGF-β induces SRC activation through a SMAD-independent, NADPH-oxidase dependent manner [109]. Specifically, TGF-β induces a transient increase in extracellular H2O2 leading to SRC activation in a redox-dependent manner which is mediated by cysteines 248, 277, 490 and 501 [109]. Moreover, Work by Park et al., showed that TGF-β-induced apoptosis is SRC-dependent. In TGF-β1 sensitive cells, TGF-β1 induces transient activation of SRC and its subsequent caspase-mediated degradation and cell death. Moreover, SRC-activation led to inhibition of apoptosis through promoting the activation of pro-apoptotic enzymes JNK, p38 and caspases [110].

#### WNT signaling

SFK activity is also important in the signal transduction of Wnt signaling pathways. Both canonical (β-catenin-dependent) and non-canonical (β-catenin independent) signaling pathways have been shown to play critical roles in the EMT in various tumors [111]. In canonical Wnt signaling, β-catenin acts as a transcriptional cofactor and its nuclear translocation upon ligand-receptor binding activates transcription of genes involved in proliferation, tumorigenesis, cell fate specification and differentiation [112]. In addition, β-catenin is a critical structural protein involved in cell–cell junctions, as previously discussed. β-catenin forms a complex with cadherins to maintain cell–cell contacts and the loss of this interactions leads to the disassembly of adherens junctions [113]. Therefore, E-cadherin is a negative regulator of the canonical Wnt pathway through sequestering of β-catenin, as all of the β-catenin available in an epithelial cell is used in the formation of adherens junctions. Activation of EMT leads to disintegration of cell–cell junctions and subsequent release of β -catenin, which transduces Wnt signaling by entering the nucleus and stimulating transcription through an association with T-cell factor (TCF) and Lymphocyte enhancer-binding factor (LEF) [114].The interaction between β-catenin and E-cadherin can be disrupted by phosphorylation of tyrosine Y654 on β-catenin, as this tyrosine is located at the site which mediates their interaction [113, 115]. In development, this interaction has been reported to be repressed during mesoderm invagination. Brunet and colleagues showed that SRC homolog, SRC42A, phosphorylates Y654 on β-catenin, which results in an increase of β-catenin in the cytoplasm and subsequent nuclear translocation [115]. The repression of SRC activity by PP2, a SRC inhibitor, resulted in the maintenance of E-cadherin/ β-catenin interaction [115]. Moreover, SRC overexpression has been implicated in regulating protein synthesis of β-catenin through a Cap-dependent mechanism [116]. Specifically, SRC enhances protein synthesis through phosphorylation of the translation initiation factors elF4E and its inhibitors elF4E-BP1 through the PI3K-mTOR and Erk-MAPK pathways [116].

In vitro studies in HER2-positive breast cancer cells showed that stimulation with recombinant WNT3A, a canonical ligand, induced expression of TWIST, SLUG and N-cadherin and repressed the expression of E-cadherin [117]. Thus, increased Wnt ligand availability promotes EMT activation and malignant progression [118–120]. Additionally, work by Gujral et al., identified an upregulation of non-canonical Wnt ligands (WNT5a and WNT5b) and their cognate receptor Frizzled2 (FZD2) in late-stage metastatic carcinomas of different origins which correlated with an increase in EMT markers and worst patient outcomes. Their work identified the WNT5a-FYN-STAT3 signaling axis as the main driver of Wnt5a-mediated cell migration and EMT [121]. Studies from our lab identified Abelson-interactor 1 (ABI1), adaptor and scaffold protein, as an EMT regulator through modulation of the Wnt5a-FYN-STAT3 axis and the WAVE regulatory complex [73]. Recent work by Villarroel and colleagues demonstrated both canonical (WNT3a) and non-canonical (WNT5a) ligands lead to activation of FYN and STAT3-mediated EMT [122]. Interestingly, ABI1 has been shown to regulate SRC kinase activity, and prevent SRC-mediated breast cancer progression [98].

#### NOTCH1 signaling

Mutliple studies have shown the link between Notch signaling and activation of the EMT through induction of SNAIL and SLUG expression in normal and pathological conditions [123–127]. Canonical Notch signaling is a type of juxtacrine cell communication, mediated by a ‘donor’ or ‘secretory’ cell and an “acceptor” or “receiver” cell [128]. Notch-1 signaling is mainly involved in controlling cell fate decisions, differentiation and proliferation. There are four isoforms of Notch [1–4] which bind to and are activated by Delta-like (DLL1, DLL3, and DLL4) or Jagged family (Jag1 and Jag2) of ligands. This binding triggers a series of proteolytic cleavage events that lead to the generation of the active, intracellular fragment of NOTCH (NICD). Notch-ICD translocates to the nucleus and associates with transcriptional activators to induce expression of target genes. Interestingly, a recent study by LaFoya and colleagues showed that SRC phosphorylates the intracellular domain of Notch at Y2074 and Y2145, which decreases NICD binding to its co-transcriptional activator Mastermind-like (MAML), impeding its transcriptional activity [128]. This suggests that in the context of NOTCH-signaling, SRC may serve as an EMT repressor.

#### EGFR signaling

The epidermal growth factor receptor (EGFR) family is a subclass of the receptor tyrosine kinase super family that is comprises of four members: ERBB1 (EGFR), ERBB2 (HER2), ERBB3 (HER3) and ERBB4 (HER4) [129]. All EGFR family members are transmembrane glycoprotein composed of an extracellular ligand-binding domain, a hydrophobic transmembrane region, an intracellular receptor tyrosine kinase (RTK) domain, and a C-terminal cytoplasmic tail [130]. EGFR members are ubiquitously expressed in various types of tissues including epithelial, mesenchymal and neuronal origin [129]. EGFR signal cascade is initiated through ligand-induced receptor dimerization which leads to activation of intrinsic tyrosine kinase (TK) domain and autophosphorylation of the cytoplasmic tail [131, 132]. Once phosphorylated, the cytoplasmic tail serve as a docking sites to recruits cellular kinases, or signal adaptor proteins for downstream signal transduction [131, 133]. Upon activation, EGFR signaling is involved in multiple cellular events like cytoskeleton dynamics reorganization and transcriptional reprograming which promote cellular activities that are critical for both maintenance of normal cell functions and malignant progression [131]. These cellular activities include proliferation, division, migration, and adhesion formation, which are determined through the specificity of different EGFR-binding ligands and the combination of EGFR dimers into homo- or heterodimers [134–141]. Enhanced EGFR signaling is often found in multi-types of epithelial oncogenic events due to overexpression or mutations of EGFR receptors, and autocrine or paracrine production of EGF family ligands [129].

In tumors, EGFR receptors have been shown to work synergistically in coordination with SRC and other tyrosine kinases, promoting tumor progression. Studies have indicated that EGFR receptors and c-SRC were co-overexpressed in 70% of breast tumors and were shown to work together to promote tumor growth in a mice xenograft study [142]. Moreover, the presence of SRC was indicated to be necessary for EGFR2-mediated anchorage-independent growth, cell motility regulation and cell survival [143–145]. The molecular mechanism of SRC/EGFR interactions have been revealed by multiple studies where SRC was shown to be associated with activated EGFR receptors, most likely via an SH2/phospho-tyrosine interaction. This leads to SRC activation, promoting the phosphorylation of its substrates [144, 146, 147]. SRC phosphorylates EGFR at Y845, which has been shown to be important for EGF-induced proliferation and survival through STAT5b and Cox II, respectively [145]. Moreover, SRC can be activated by other signaling cascades like G-protein coupled receptors (GPCRs) to promote phosphorylation of multiple tyrosine sites of EGFR receptors, including Tyr 845 [148–150]. Other studies have indicated that through phosphorylation of clathrin and dynamin, SRC modulates the internalization of EGFRs to enhance the endosomal pool of activated receptors [151, 152]. SRC interacts with EGFR to promote degradation of Cbl, a E3 ubiquitin-protein ligase, by SRC-mediated Cbl phosphorylation, which protects EGFR receptors from Cbl mediated ubiquitination and degradation, allowing for receptor recycling [153]. Due to the critical oncogenic roles of EGFR signaling, various EGFR tyrosine kinase inhibitors (TKI) have been developed to abrogate EGFR signaling by competing with ligand binding or the ATP binding pocket on the catalytic domain [130]. However, treatment resistance emerges due to secondary EGFR mutations and/or activation of alternative pathways [154–156].

## SFK’s role in mediating treatment resistance in targeted-therapies

Metastatic disease accounts for over 90% of cancer-related deaths which underscores the importance in understanding key cellular pathways involved in mediating treatment resistance [157, 158]. Both SFKs and EMT activation have been identified as key mediators of treatment resistance to a variety of different anti-cancer therapies [159–161]. Concurrent pan-SRC inhibition has been shown to restore sensitivity to a variety of therapies which underscores the importance of SFK in mediating treatment resistance [162–165]. However, while pre-clinical studies have suggested great promise in the use of SFK-inhibitors, their clinical outcomes have left much to be desired. Therefore, in this segment we will evaluate mechanisms through which SFKs have been implicated in mediating treatment resistance and its implications in the treatment of metastatic disease. To generate a more thorough review, we will focus on treatments targeting the previously mentioned pathways for which SFK activity is of critical importance (see “Src family kinases in the epithelial mesenchymal transition (EMT)” section).

### SFK’s role in mediating treatment resistance in targeted-therapies

In breast cancer, SRC activation is associated with overexpression of P-cadherin and stem cell-like phenotype that promotes cell invasion and tumorigenesis. SRC inhibition by dasatinib disrupts P-cadherin downstream signaling, rescues cell membrane E-cadherin/p120-catenin complex and recovers cell–cell adhesion properties [166]. Several studies have addressed the interactions and cross-talks among SRC, ER and AR signaling pathways. SRC activation is observed in ~ 40% of ER + breast cancer and synergistic interaction between EGFR, ER and SRC facilitates hormone receptor signaling and confers resistance to endocrine therapies [167]. Moreover, SRC activation has been shown to contribute to tamoxifen resistance in pre-clinical models and is associated with a poor patient response to tamoxifen clinically [168]. Later work by Vallabhaneni and colleagues showed that in the context of proline, glutamic acid, leucine-rich protein 1 (PELP1), an ERα co-regulator and proto-oncogene, SRC inhibition could overcome endocrine resistance [169]. A different study suggests that LYN activity could mediate anti-estrogen therapy resistance in estrogen receptor-positive (ER( +)) breast cancers [170]. Work by Elias, et al., in breast cancer also identified FYN as an important molecule in tamoxifen resistance [171]. Specifically, their study suggested that through phosphorylation of cell cycle proteins such as Cdc25A, FYN was helping to overcome the anti-proliferative effects of tamoxifen [171]. However, it is still not clear how different members of SFK family are involved in cancer progression and how distinct SFKs-dependent molecular mechanisms regulate tumorigenic pathways in different cancer types. Tabaries et al. bring attention to the opposing roles of different SFK family members in breast cancer and advocate for the use of selective inhibitors in clinical practice. Their study shows that Claudin 2—dependent breast cancer metastasis is differentially regulated by different SFKs. Their in vivo studies showed loss of YES or FYN induces Claudin-2 expression; whereas, diminishing LYN levels impairs Claudin-2 expression and reduces breast cancer metastasis [172]. Pro-metastatic activity of LYN has also been suggested in Ewing’s sarcoma by a study showing a significantly decreased tumor invasive capacity in cell culture when LYN was inhibited by small interfering RNA [173].

Another class of targeted therapy which has played a critical role in the treatment of metastatic breast cancer is receptor tyrosine kinase inhibitors such as trastuzumab, which targets the human epidermal growth factor receptor 2 (HER2). However, resistance to therapy is common and SRC activation has been linked to resistance to anti-HER2 therapy [174]. HER2 associates with SRC and promotes SRC activation through promoting its synthesis and stability. One of the important anti-tumor mechanisms of trastuzumab is inhibition of HER2-mediated SRC activation, thus SRC remains inactive and unable to inhibit phosphatase and tensin homolog (PTEN), leading to its reactivation as a functional tumor suppressor. However, SRC signaling is often upregulated in breast tumors as a result of many different upstream dysregulations (i.e. RTK re-programming and PTEN loss) and does not require HER2 for activation. This leads to resistance to anti-HER2 therapy in a SRC-dependent manner [175, 176]. Another mechanism of SRC-mediated trastuzumab-resistance was reported by Liang and colleagues which involves EpoR, a receptor co-expressed with HER2 in breast cancer cells. Ligand-engagement of the EpoR leads to JAK2-mediated SRC activation [177]. Moreover, TGFβ integrates HER2 receptor and integrin signaling leading to SRC-FAK pathway activation and trastuzumab resistance [178]. In addition to trastuzumab, acquired-resistance to other RTK-targeted therapies (i.e. lapatinib) have been reported to involve SRC hyperactivation [176]. Convergence of these resistance mechanisms on SRC led to studies by Zhang et al., which showed addition of a SRC inhibitor to trastuzumab-resistant breast cancer cell lines restored sensitivity to trastuzumab therapy in vivo [174].

Similar to breast tissue, prostate tissue is responsive and dependent on hormonal-stimulation for growth—specifically it is dependent on androgens. The androgen receptor (AR) is a nuclear hormone receptor that regulates gene expression of proteins involved in cell proliferation and tumor growth. AR signaling is commonly upregulated in prostate cancer and has been associated with inducing EMT [179]. Guo and colleagues found that there was an elevated tyrosine phosphorylation at Y534 of AR in hormone refractory tumor samples, associated with increased nuclear translocation and transcription factor activity in androgen depleted conditions [106]. Work by Whang and colleagues found that SRC-mediated phosphorylation of AR at tyrosine Y534 could promote androgen independent growth in hormone dependent cell lines [105, 180]. Together these data suggests that SRC-mediated phosphorylation of the AR plays a role in mediating cancer progression and development of treatment resistance. Furthermore, genomic profiling studies of prostate cancer cell lines demonstrated an inverse correlation between SRC activity and androgen signaling, implying that SRC activity plays a role in endocrine resistant prostate tumors which was shown to be overcome with SRC inhibition [181]. Moreover, in a phase-II study dasatinib monotherapy, a pan-SFK inhibitor, seemed promising for the treatment of chemotherapy-naïve, metastatic castration-resistant prostate cancer (mCRPC) with bone metastasis [182]. However, its efficacy was diminished when used for treatment of mCRPC in patients who had undergone prior chemotherapy regimens [183].

SRC-mediated resistance to RTK-targeted therapies has also been observed with anti-EGFR therapies. EGFR and SRC activities are elevated in the majority of the lung, colorectal and pancreatic tumors. EGFR interacts with SRC, and the transformation capability of two EGFR catalytic domain mutants has been shown to be dependent on SRC [156]. SRC activation enhances EGFR activation and downstream PI3K/Akt signaling, thus SRC has been suggested to mediate cetuximab resistance through facilitating nuclear translocation of EGFR. The combination of dasatinib with erlotinib, an EGFR inhibitor, showed more promising results with inhibition of angiogenesis and eliciting disease control in 63% of patients [184]. In the analysis of combined erotinib, dasatinib treatment of patients with head and neck squamous cell carcinoma (HNSCC), it has been suggested that basal expression of pSTAT3 may be independent of SRC and explain therapeutic resistance to dasatinib [185]. Dasatinib in combination with cetuximab, an EGF inhibitor, has been demonstrated to be safe and has a potential to overcome cetuximab resistance in solid tumors [186, 187]. Furthermore, a study by Weng et al., showed the mesenchymal phenotypes of TKI-resistant cells, suggesting the EMT contributes to the acquired resistance of EGFR TKI treatments [188]. In this study, authors showed the constitutive activation of EGFR signaling in TKI-resistance cells renders EMT features in cells through activation of the SRC/Hakai axis, which mediates E-cadherin ubiquitination and degradation [188, 189]. They further indicated that the inhibition of SRC/Hakai axis reversed EMT phenotypes by stabilizing cellular E-cadherin levels, which increases the sensitivity to TKI in TKI-resistance cells. This underscores the importance of SRC-mediated EMT processes in inducing treatment resistance of targeted therapies.

### Lessons learned from Src inhibitors

Despite large preclinical data that demonstrated therapeutic promise of SFK inhibition in different tumors, so far there have not been much success demonstrated in clinical trials using SFK inhibitors as monotherapy. Current FDA-approved SFK inhibitors include bosutinib, dasatinib, and ponatinib for the treatment of chronic myelogenous leukemia and vandetanib for the treatment of medullary thyroid cancer. However, these inhibitors lack specificity among SFK members and they are known to inhibit other tyrosine kinases [9]. Dasatinib monotherapy did not appear to be promising in patients with metastatic non-small cell lung carcinoma (NSCLC) with clinical activity lower than what is generally observed in patients who receive chemotherapy [190, 191]. Moreover, limited single-agent activity with dasatinib was also observed in patients with advanced HR + breast cancer [192]. The SrRC and BCR-Abl inhibitors saracatinib and AZD0424 that showed promise in pre-clinical studies also have not demonstrated expected efficacy in patients. In relapse clear cell renal cell carcinoma, advanced pancreatic adenocarcinoma, and ovarian, fallopian-tube and peritoneal cancer, addition of Saracatinib did not increase the efficacy of standard therapy [193–195]. AZD0424 displayed no evidence of efficacy as monotherapy despite a clear pharmacodynamic effect [196]. However, it is important to consider that activation of alternative oncogenic pathways which lead to diminish SRC signaling may render resistance to SRC inhibitors. For example, the status of the von Hippel-Linday (VHL) gene in renal cell carcinoma dictates sensitivity to SRC inhibition through the HIF-regulated VHL-PTP1B-SRC signaling axis [197]. Moreover, studies by Matrone and colleagues showed that SRC inhibition leads to promotion of microtentacle formation, a microtubule-based protrusion involved in capillary retention of circulating tumor cells to distant organ sites [198]. This suggests monotherapy with SFK-inhibitors may promote rather than inhibit metastatic disease. This underscores the importance of developing SFK-inhibitors with higher specificity, as lack of selectivity may select for more invasive malignancies.

It has been suggested that the efficacy of clinical trials has been complicated by the patient population, with heavily penetrated tumors resistant to previous therapies and as well as by the absence of definite biomarkers that could suggest therapeutic effectiveness [199, 200]. The lack of definitive biomarkers was previously addressed by Arcaroli and colleagues, whose work showed that SRC pathway activation was a good indicator of sensitivity to SRC inhibition [201]. However, this has not been implemented in the design of clinical trials accessing the efficacy of SFK inhibitors in metastatic disease.

The synthetic lethality approaches that are now being used more and more in the treatment of cancer could be applied to the issue of complementation of SFK in signaling and where FYN and YES, for example, are upregulated in response to decreased SRC activity. As previously discussed, SFKs function as key connectors of proliferation and survival pathways and often serve to promote resistance to a variety of targeted therapies. Therefore, in addition to currently available inhibitors which target the kinase activity of SFKs, it would be imperative to develop SH2 or SH3 inhibitors which target protein interactions of SFKs with the goal to minimize compensation and crosstalk between signaling pathways. These type of inhibitors could potentially decrease acquire-treatment resistance due to activation of alternative pathways mediated by SFK activation.

## Conclusion

SFK’s role in invasion, metastasis, angiogenesis, tumor-microenvironment, immune-modulation and many other cellular processes makes it clear that while SFK-dysregulation may not always lead to activation of EMT, EMT requires SFK activity for execution. Metastatic carcinomas are challenging to treat due to their ability to activate rapidly evolving programs which renders them adaptable to intrinsic and extrinsic insults such as the tumor microenvironment and anti-cancer treatments. Partial responses to treatments generate a race between host stromal and carcinoma cells, in which natural selection takes its course. Only those carcinoma cells “well-adapted enough” to the new TME will survive, proliferate and continue to invade and metastasize. It is thus critical to evaluate the molecular changes induced by single and combination therapies on the cellular pathways, to assess for alterations which may serve as prognostic markers and/or therapeuric targets. As reviewed here, we see that members of the Src protein family share many similarities, however, there are key differences which could be utilized to magnify their individuality and generate inhibitors with higher specificity. As key connectors of signal transduction SFK activity is altered through many pathways such as those involved in intracellular, intercellular, and cell-ECM interactions without the need for alteration in total protein expression nor function. Moreover, it is clear that while EMT-TFs are detailing out the plan, SFKs belong to the front-line fighters which execute these plan. Therefore, it is imperative to continue to evaluate the role of individual SFKs in metastatic progression to be able to address the much needed clinical need for treatments of metastatic disease.

## Data Availability

Not Applicable.

## Abbreviations

ABI1: Abelson interactor 1
AJ: Adherens junctions
AR: Androgen receptor
ARP2/3: Actin-related protein 2/3
ATP: Adenosine triphosphate
BLK: B lymphoid tyrosine kinase
BRK1: Breast tumor kinase
CRB: Crumbs
CSC: Cancer stem cells
CYFIP: Cytoplasmic FMRP Interacting Protein 1
DLG: Discs large
DLL: Delta-like ligand
EGFR: Epidermal growth factor receptor
EMT: Epithelial to mesenchymal transition
EMT-TFs: EMT transcription factors
EpoR: Erythropoietin Receptor
ER: Estrogen receptor
FA: Focal adhesion
FAK: Focal adhesion kinase
FDA: Food drug administration
FGR: Feline sarcoma viral protein Gardner-Rasheed
FZD2: Frizzled2
HCK: Hemopoietic cell kinase
HER: Human epithelial receptor
HIF: Hypoxia inducible factor
HR: Hormone receptor
IDRs: Intrinsically disordered regions
JAK2: Janus kinase 2
JNK: C-Jun N-terminal kinase
LCK: Lymphocyte cell-specific protein tyrosine kinase
LEF: Lymphocyte enhancer-binding factor
LGL: Lethal giant larvae
LYN: Lck-related novel protein tyrosine kinase
MAML: Mastermind-like
MAPK: Mitogen activated protein kinase
MET: Mesenchymal to epithelial transition
MMP: Matrix metalloproteinases
mTOR: Mammalian target of rapamycin
N-WASP: Neural Wiskott–Aldrich Syndrome protein
NADPH: Nicotinamide adenine dinucleotide phosphate
NAP: Nucleosome assembly protein
NICD: Intracellular fragment of NOTCH
NMDARs: N-methyl D-aspartate receptors
NOTCH: Notch homolog 1
NPF: Nucleation-promoting factors
NSCLC: Non-small cell lung carcinoma
PALS1: Protein-associated with Lin-7 1
PAR: Partitioning-defective
PCK: Protein kinase C
PDGFR: Platelet-derived growth factor receptor
PI3K: Phosphoinositide 3-kinase
PSD: Post-synaptic densities
PTP1B: Protein tyrosine phosphatase 1B
RTK: Receptor tyrosine kinase
SCRIB: Scribble
SFK: Src family kinases
SH: Src homology
Shp1/2: Src homology region 2 domain-containing phosphatase-1/2
SLUG (SNAI2): Snail homolog 2 of drosophila
SMAD: An acronym from the fusion of Caenorhabditis elegans Sma genes and the Drosophila Mad, Mothers against decapentaplegic
SNAIL: Snail homolog 1 of drosophila
SRC: Sarcoma
STAT: Signal transducer and activator of transcription
TCF: T-cell factor
TGF-β: Transforming growth factor beta
TIMP2: Tissue inhibitor of metalloproteinases 2
TK: Tyrosine kinase
TKI: Tyrosine kinase inhibitors
TWIST: Twist homolog 1 of drosophila
UD: Unique domain
ULBR: Unique lipid binding region
v-SRC: Viral avian SRC
VHL: Von Hippel-Linday (VHL)
WASP: Wiskott–Aldrich syndrome protein
WAVE: WASP-family verprolin-homologous protein
WNT: Wingless/integrated
WRC: WAVE regulatory complex
ZEB: Zinc finger E-box binding homeobox
